# Epigenome wide association study of response to methotrexate in early rheumatoid arthritis patients

**DOI:** 10.1371/journal.pone.0247709

**Published:** 2021-03-10

**Authors:** Helen R. Gosselt, Costanza L. Vallerga, Pooja R. Mandaviya, Erik Lubberts, Johanna M. W. Hazes, Robert de Jonge, Sandra G. Heil

**Affiliations:** 1 Department of Clinical Chemistry, Erasmus MC, University Medical Center Rotterdam, Rotterdam, The Netherlands; 2 Department of Clinical Chemistry, Amsterdam Gastroenterology and Metabolism, Amsterdam UMC, Vrije Universiteit Amsterdam, Amsterdam, The Netherlands; 3 Department of Internal Medicine, Erasmus MC, University Medical Center Rotterdam, Rotterdam, The Netherlands; 4 Department of Rheumatology, Erasmus MC, University Medical Center Rotterdam, Rotterdam, The Netherlands; 5 Academic Center of Excellence − Inflammunity, Erasmus MC, University Medical Center Rotterdam, Rotterdam, The Netherlands; Massachusetts General Hospital, UNITED STATES

## Abstract

**Aim:**

To identify differentially methylated positions (DMPs) and regions (DMRs) that predict response to Methotrexate (MTX) in early rheumatoid arthritis (RA) patients.

**Materials and methods:**

DNA from baseline peripheral blood mononuclear cells was extracted from 72 RA patients. DNA methylation, quantified using the Infinium MethylationEPIC, was assessed in relation to response to MTX (combination) therapy over the first 3 months.

**Results:**

Baseline DMPs associated with response were identified; including hits previously described in RA. Additionally, 1309 DMR regions were observed. However, none of these findings were genome-wide significant. Likewise, no specific pathways were related to response, nor could we replicate associations with previously identified DMPs.

**Conclusion:**

No baseline genome-wide significant differences were identified as biomarker for MTX (combination) therapy response; hence meta-analyses are required.

## Introduction

Methotrexate (MTX) is currently the anchor drug in the treatment of rheumatoid arthritis (RA), in agreement with the recommendations of the European league against rheumatism (EULAR)/ American college of rheumatology (ACR) [1]. However, treatment strategies are still trial and error due to the fact that treatment response is unknown until 3 to 6 months from initiation. While, about 30–40% of patients do not benefit from MTX. Clearly, there is a need for biomarkers to predict response prior to treatment in order to enable tight control of disease activity within the ‘window of opportunity’ and to restrain radiographic joint damage and functional disability [2].

Genomic DNA methylation at CpG dinucleotides has previously been associated with disease onset of RA [3–6] and therefore could possibly be utilized as a predictor for response to treatment with MTX (combination) therapy. Previously, we found an association between global DNA methylation and response to MTX in RA [7]. Other studies examined the relationship between differentially methylated positions (DMPs) in blood cells and DAS28 using Illumina’s HumanMethylation450 BeadChip [8, 9]. While Glossop *et al*. described that a combination of methylation levels at cg03018489 and cg14345882 in T-lymphocytes at baseline best predicts response to DMARD therapy at 6 months according to the EULAR criteria [8], Nair *et al*. did not find significant associations at baseline with changes in DAS28 over 6 months [9]. However, in the latter study, 4 DMPs at 4 weeks were associated with changes in single DAS28 components, such as swollen joint count and c–reactive protein, over 6 months [9]. Since a few years, Illumina has made available a new DNA methylation platform, the HumanMethylationEPICBeadChip array including >850,000 probes. This newly designed array is an extension of the Illumina HumanMethylation450 BeadChip, covering ~ 90% of previous sites and over 400,000 new probes of which the majority is positioned in potential enhancers [10].

In this study, we examine differentially methylated positions and regions in treatment naïve early RA patients in relation to treatment response to MTX assessed over the first 3 months of treatment initiation.

## Materials and methods

### Patients and materials

Patients were included from the treatment in early arthritis cohort Rotterdam (tREACH, registration number: ISRCTN26791028), a multicenter stratified single-blinded clinical trial of early rheumatoid arthritis (RA) patients [6]. Patient inclusion for current study was based on the availability of baseline Peripheral Blood Mononuclear Cells (PBMCs), which resulted in the inclusion of 83 patients. The tREACH was described earlier [6]. In short, inclusion criteria for the tREACH were the presence of arthritis in one or more joint(s), age ≥18 years and symptom duration < 1 year. This study was approved by the medical ethics committee of the Erasmus MC, University Medical Center Rotterdam (MEC-2006-252). Medical ethics committees at each participating center (Erasmus MC, University Medical Center, Rotterdam; Sint Franciscus Gasthuis, Rotterdam; Maasstad Ziekenhuis, Rotterdam; Vlietland Ziekenhuis, Schiedam; Admiraal de Ruyter Ziekenhuis, Goes and Vlissingen; Zorgsaam Ziekenhuis, Terneuzen; Albert Schweitzer Ziekenhuis, Dordrecht) approved the study protocol and written informed consent was obtained for all including patients. Patients were recruited between July 2007 and April 2014 from outpatient clinics in participating centers in and near Rotterdam. The research for this manuscript took place in the Erasmus MC University Medical Center, Rotterdam. For current study, at baseline all patients were treatment naïve and were randomized to start methotrexate with corticosteroids as monotherapy or in combination with other disease modifying antirheumatic drugs (DMARDs): sulfasalazine (SSZ) and hydroxychlorqiuine (HCQ). In the tREACH, MTX dose was quickly increased (from 10 mg to 25 mg/week) within the first 3 weeks. Due to this aggressive treatment strategy in the tREACH, early response rates were expected at 3 months. If the target low disease activity (DAS28 <3.2) at 3 months was not reached, step-up treatment with biological or targeted synthetic DMARDs was prescribed. Additionally, patients weekly received 10 mg folic acid to reduce adverse events. PBMCs were extracted from whole blood using BD vacutainer ^®^ CPT and stored in Roswell Park Memorial Institute (RPMI) 1640 Medium (R0883, with sodium bicarbonate, without L-glutamine, Merck) and 10% dimethylsulfoxide in liquid nitrogen.

### DNA extraction

DNA extraction was performed for these 83 subjects using AllPrep DNA/RNA mini kit (Qiagen, Hilden, Germany) for simultaneous DNA and RNA isolation with a minimum input of 1 x 10^5^ cells. DNA concentrations were assessed using a Nanodrop (NanoDrop Technologies, Wilmington, Germany). 72 samples with 260/280 ratios between 1.7 and 2.0 and of at least 500 ng were included in further analysis.

### Human methylation EPIC BeadChip

72 samples of 500 ng DNA were bisulfite treated using the Zymo EZ-96 DNA methylation kit (Zymo Research, Irvine, CA, USA). DNA methylation was quantified using the Infinium Human Methylation EPIC BeadChip Array according to manufacturer’s protocol (Illumina, Inc., San Diego, CA, USA). Quality control and normalization was performed in R according to the incorporating Control Probe Adjustment and reduction of global CORrelation (CPACOR) workflow, as described previously [11]. In short, intensity values were stratified to autosomal and non-autosomal probes followed by quantile normalization for the six probe type categories separately (type I methylated red/green, type I unmethylated red/green and type II red/green). Beta values were calculated as a ratio between the fluorescence intensities of the methylated (M) and the M + unmethylated (U) probe intensity + a constant as follows: beta value = M/(M+U+100). Beta values below the first quartile—1.5 x interquartile range (IQR) or above the third quartile + 1.5 x IQR were considered outliers and were set to missing. Three samples did not pass quality control (N = 2 due to unsuccessful bisulfite conversion, and N = 1 due to unsuccessful hybridization) and were excluded. No gender mismatches or sample call rates below 98% were identified. Furthermore, probes with an intensity detection p-value ≥ 10^−16^ in > 5% of the samples were removed (N = 14,184). The final dataset contained 846,415 probes and 69 patients.

### Gene annotation

CpGs were annotated using the Illumina annotation file Version B.04. Missing gene names from the file were replaced by annotations using the Genomic Regions Enrichment of Annotations Tool (GREAT) (Human GRCh37 UCSC hg19, Feb/2009), where nearest basal regulatory regions within 5 kb upstream and 1 kb downstream of the transcription start site (TSS) with a maximum up to 1 MB were considered.

### Epigenome-Wide Association Study (EWAS)

Associations between baseline differentially methylated positions (DMP) and changes in disease activity over the first three months (ΔDAS28) were examined using MOMENT; a mixed-linear-model-based method using OmicS-data-based Complex train Analysis (OSCA) software [12]. This method tests for associations between baseline methylation and the linear outcome: ΔDAS28 and fits all distal probes in multiple random-effect components to account for unobserved confounders resulting in fewer false positive rates than other methods [12]. Prior to analysis, all beta values and the outcome were standardized to improve the comparison of effect sizes across probes. In our model the outcome was linear ΔDAS28 (i.e. 3 months DAS28 –baseline DAS28) as response rates were expected within 3 months due to the study design of the tREACH. We corrected for cell type composition using the Houseman method [13]. The association analysis in treatment naïve PBMCs was adjusted for the following covariates: baseline DAS28, gender, age, smoking and cell type composition (Houseman predicted: CD4T lymphocytes, CD8T lymphocytes, B lymphocytes, natural killer cells and monocytes). The smoking status of individuals included in our study was predicted using methylation profiles of targeted CpG sites known to be strongly associated with smoking, using the ‘EpiSmokEr’ package in R [14]. In addition, batch effects (plate and position) were treated as random effects to adjust for technical biases. Furthermore, MTX-polyglutamate concentrations that were previously determined in tREACH erythrocyte cell pellets [15] were used to assess treatment compliance in this study. With the aim of increasing the power of our study and with the rational that big differences between response groups would not be observed in probes with low biological methylation variance [16], association analyses were repeated filtering out lowly variable probes (probes with baseline methylation standard deviation <0.02) and excluding sex chromosomes. All tests were adjusted for multiple comparisons using Bonferroni correction.

### Differentially methylated regions

To examine whether probes that lay in the same epigenomic region show the same relation to response to MTX (combination) therapy, differentially methylated regions (DMR) analyses were performed. DMRs were assessed using the DMRff package, which has been shown to be robust and control well for false positive rates [17]. Standard parameters of the DMRff package were applied to define genomic regions: at least two CpGs had to be present to form a region, the distance between probes within a DMR was maximum 500 base pairs, additionally CpGs had nominal EWAS p-values <0.05 and effect estimates of probes within a DMR were in the same direction [17].

### Pathway analysis

Explorative gene ontology (GO) term and Kyoto Encyclopedia of Genes and Genomes (KEGG) pathway analysis were performed using the top 1000 probes of DMP results, if nominal DMP p−values were <0.05, using the ‘missMethyl’ package in R.

## Results

### Patient characteristics

14,184 probes out of 860,599 were removed during quality control. Hereafter, 69 subjects and 846,415 probes were included. The majority of patients were female (58%) with a mean age of 50.6 ± 15.4 years (Table 1). Mean DAS28 at baseline was 4.8 ± 1.3. 79.7% of the patients were positive for anti-citrullinated protein antibody (ACPA) and 82.6% were positive for rheumatoid factor. All patients received methotrexate with corticosteroids, of which 59.4% additionally received SSZ and HCQ (Table 1). Mean ± SD response determined over three months (ΔDAS28) was not significantly different for different treatment groups (High A: -1.9 ± 1.3, High B: -1.9 ± 1.1, High C: -1.8 ± 1.1, p = 0.963). Smoking status could not be determined for 1 subject; hence 68 subjects were included in the analysis.

**Table 1:** Baseline characteristics of subjects in the study.

	Mean ± SD
**Subjects, N**	69
**Sex, Female (%)**	40 (58.0)
**Age (years)**	50.6 (15.4)
**Baseline DAS28**	4.8 (1.3)
**BMI (kg/m**^**2**^**)**	26.8 (5.3) *
**ACPA positive, N (%)**	55 (79.7)
**RF positive, N (%)**	57 (82.6)
**Smoking score, median (IQR)**	3.5 (1.3)
**Treatment, N (%)**	
**MTX + SSZ + HCQ + i.m. corticosteroids**	21 (30.4)
**MTX + SSZ + HCQ + p.o. corticosteroids**	20 (29.0)
**MTX + p.o. corticosteroids**	28 (40.6)

*BMI: 1 missing value. MTX = methotrexate, SSZ = sulfasalazine, HCQ = hydroxychloroquine, i.m. = inter muscular, p.o. = per os. A smoking score was calculated using the EpiSmokEr package in R.

### Analysis complete probe set

**Fig 1:**
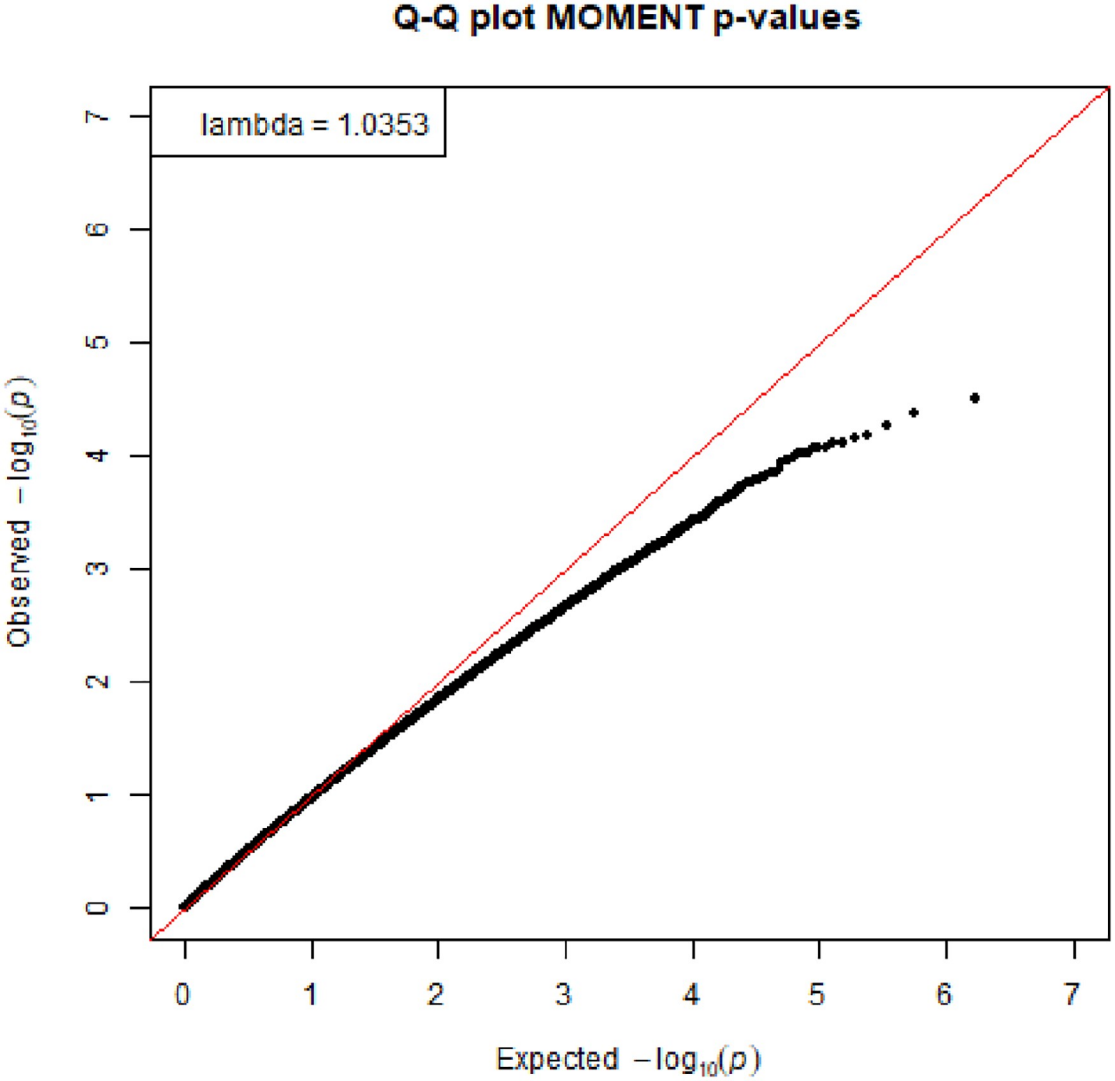
QQ-plot of linear mixed model for the association of DMPs and ΔDAS28.

**Fig 2:**
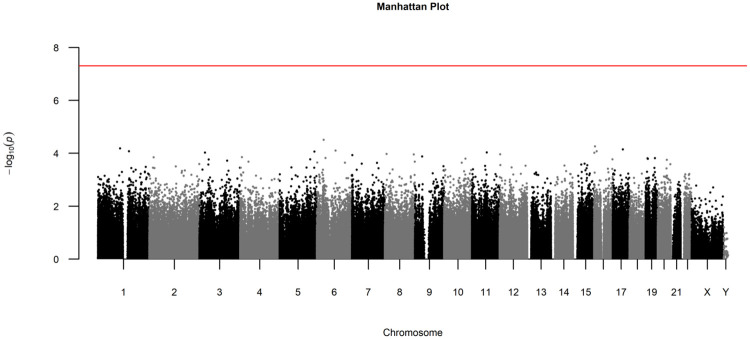
Manhattan plot of DMP analysis with all probes in association with ΔDAS28. Associations were adjusted for age, gender, smoking and cell type ratios. The red line indicates genome-wide significance according to Bonferroni correction (p-value = 5.91^−8^).

The association between DMPs at baseline and changes in DAS28 over the first three months was assessed in a linear mixed model corrected for baseline DAS28, age, gender, smoking status and cell type composition. The Quantile-Quantile (QQ) plot with corresponding lambda, a measure to quantify the inflation in the test statistic, is shown in Fig 1. We did not observe genome-wide significant differences (0.05/846,415 = 5.9 x 10^−8^) nor DMPs located in certain chromosomes, as can be seen from the Manhattan plot (Fig 2). The top 10 DMPs with nominal p-values ≤ 1.0 x 10^−4^ are presented in Table 2.

**Table 2:** Top 10 DMP results from the analysis with complete probe set and the association with ΔDAS28.

Probe	Chr	Position	Gene	Relation to gene	450k loci	β	se	p
**cg16944926**	6	32940976	*BRD2*	Body	Yes	0.42	0.10	3.09 x 10^−05^
**cg11177738**	3	193828742	*HES1*, OPA1*	-	No	0.45	0.11	4.42 x 10^−05^
**cg00519627**	16	4466650	*CORO7*	TSS200	Yes	-0.45	0.11	5.43 x 10^−05^
**cg15697822**	1	107684751	*NTNG1*	5’UTR	Yes	0.41	0.10	6.54 x 10^−05^
**cg02802788**	17	49369718	*UTP18*	Body	No	-0.39	0.10	7.09 x 10^−05^
**cg14665002**	12	64919341	*RASSF3*, TBK1*	-	No	0.40	0.10	7.80 x 10^−05^
**cg07248935**	6	90643780	*BACH2*	Body	No	0.68	0.17	7.81 x 10^−05^
**cg11311263**	16	11829117	*TXNDC11*	Body	No	-0.40	0.10	8.31 x 10^−05^
**cg00095674**	1	150122654	*PLEKHO1*	Body	Yes	-0.42	0.11	8.36 x 10^−05^
**cg26426470**	5	169181253	*DOCK2*	Body	Yes	-0.39	0.10	8.60 x 10^−05^

β = standardized beta coefficient.

*Genes annotated using the GREAT tool (Human GRCh37 UCSC hg19). Chr = chromosomes, TSS200 = 0–200 bases from transcriptional start site (TSS), TSS1500 = 200–1500 bp from TSS. Relation to gene was obtained from the Illumina annotation file version B4, a dash means that probes were not related to a gene by Illumina.

All adjusted p-values for multiple comparisons were ≥0.985. Results of the DMP analysis were used as input for the DMR analysis to examine whether closely located probes in certain regions show the same effect sizes and directions. We identified 1309 DMR regions, of which none were genome-wide significant. The top 10 DMRs at nominal p-values < 1.0 x 10^−3^ are presented in Table 3. Additionally, to examine if certain Gene Ontology (GO) terms were enriched, pathway analysis was performed on the top 1000 most significant DMP results, all at nominal p-values <2.5 x 10^−3^ and adjusted p-values of ≥ 0.960. The top 10 identified GO terms are presented in S1 Table, however, no genome-wide significantly enriched pathways were observed.

**Table 3:** Top 10 DMR results with complete probe set.

Chr	start	end	Nearest gene (distance to TSS)	N	b	se	p-value
8	**87355594**	87355773	*WWP1* (+717)	3	-0.40	0.10	1.05 x 10^−04^
22	**41252959**	41253041	*XPNPEP3* (-102)	3	-0.31	0.08	1.17 x 10^−04^
19	**50380748**	50380763	*TBC1D17* (+74)	2	-0.49	0.13	1.24 x 10^−04^
7	**1610694**	1610747	*PSMG3* (-1092)	2	-0.06	0.02	1.35 x 10^−04^
9	**99540409**	99540427	*ZNF510* (-80)	3	-0.44	0.11	1.39 x 10^−04^
4	**59850015**	59850171	None	2	0.18	0.05	1.42 x 10^−04^
6	**32829062**	32829208	*PSMB9* (+7197)	3	-0.14	0.04	1.47 x 10^−04^
3	**49157911**	49158377	*USP19* (+120)	6	-0.33	0.09	1.64 x 10^−04^
2	**98262546**	98262568	*COX5B* (+54)	2	-1.05	0.28	1.72 x 10^−04^
3	**45017855**	45017955	*ZDHHC3* (-231)	2	-0.72	0.19	1.90 x 10^−04^

Chr = chromosome, N = number of probes within DMR, b = change in DAS28 upon 1% difference in baseline methylation.

### Analysis filtered probes

To increase power to detect significantly associated probes with treatment response, association analysis was also carried out on a restricted set of probes (N = 393,282), after the removal of low variance probes and probes on sex chromosomes. Despite the strong reduction in the number of tested probes, we could not identify probes with genome-wide significance (0.05/393,282 = 1.3 x 10^−7^). Manhattan and QQ-plots are presented in S1 and S2 Figs. The top 10 DMPs that reached nominal significance of p< 1.82 x 10^−04^ are presented in S2 Table. DMR analysis did not find genome-wide significant regions, however 359 nominally significant candidate regions were identified, of which the top 10 regions had a nominal p-value <1.0 x 10^−3^ (S3 Table).

### Look up of previously identified loci

A study by Glossop et al described two CpGs (cg03018489 and cg14345882) in T lymphocytes of treatment naïve rheumatoid arthritis patients that could discriminate non-responder and moderate/good responders at baseline with an area under the curve of 0.85. We did a look up of these CpGs in our study. Cg03018489 was removed during the quality control steps in our study and could therefore not be assessed. Mean DNA methylation of cg14345882 was similar across good responders (mean = 0.23, sd = 0.09), moderate responders (mean = 0.23, sd = 0.09) and non-responders (mean = 0.19, sd = 0.06) as depicted in S3 Fig.

## Discussion

We present the first study that assesses baseline differential DNA methylation in relation to DAS28 in rheumatoid arthritis (RA) patients using Illumina’s Human Methylation EPIC array. In this study, we did not identify genome-wide significant DMPs or DMRs in relation to changes in DAS28 over the first 3 months of treatment. However, some of the genes with p-values of <10^−4^ have previously been associated with RA. Examples include *BRD2* [18], which binds to IL-6 promoters in macrophages where it stimulates IL-6 production, *PLEKHO1*, which regulates joint inflammation [19] and *BACH2* [20–22] and *DOCK2* [23, 24] which are important in B cell differentiation and T cell regulation, both important events in the development of early RA. These probes are therefore interesting targets for future studies.

To examine whether the top 10 most significant probes in DMP analysis were part of a DMR, we compared the top 10 DMP and DMR results. However, we did not observe any overlap between the top 10 most significant findings. Moreover, explorative pathway analysis was performed on the top 1000 results, which did not suggest a specific pathway that was differentially methylated in relation to MTX (combination) therapy response. Importantly, the results from our pathway analysis should be interpreted with care, as the top 1000 DMP results were not genome-wide significant.

Furthermore, a look up of previously described baseline CpGs related to prediction of response to MTX did not show similar results in our data. These results may reflect differences in cell types assessed [8]. Our results were from a cell mixture, hence T cell specific differences that were previously observed could have been underestimated in our results. Another explanation could be that we show larger biological variance as our intermediate (n = 28) and good responder groups (n = 31) were slightly larger compared to the study of Glossop *et al*.

Next, we repeated our association analysis by filtering out low variance probes and probes on sex chromosomes as we postulated that these probes are expected to be less informative and their exclusion could increase the power of our study. This resulted in a shift of the top findings. Most significant hits in previous DMP analysis appeared to have been in lowly variable probes as they were no longer present after filtering. Only one of the previous top 10 findings (probe cg07248935 located in the *BACH2* gene) remained in the top 10 CpGs of this second EWAS. Genes listed in top 10 most significant DMPs in the second EWAS were all part of the top 30 most significant results of the first EWAS: including all the probes. As expected, effect sizes between the two EWAS studies were similar. Upon exclusion of lowly variable probes, probe cg07639783 in the top 10 DMP list, located in *PSMG3* promoter, overlapped with a top 10 most significant DMR regions. This region was also observed in the top 10 DMR analyses with all probes. This supports that is could be an interesting target. It should however be noted that this region only consisted of two DMPs and this finding was not genome-wide significant. Hence, this potential finding should be further investigated in other studies.

On the one hand, the observation that the most significant findings were found for lowly variable probes may indicate false positive results due to the small biological variance (SD < 0.02). On the other hand, differences in methylation related to RA response have been shown to be small; hence our findings may still be clinically relevant. To find genome-wide significant results for small differences (2%) in case–control studies, very large sample sizes (>1000) are required [25]. We calculated the power of our study based upon two equal groups and this showed that we had 80% power to detect a mean difference of 8% in 77.5% of all genomic sites with recommended significance threshold of 9.42 x 10^−8^. If we assume that the power calculation for two equal groups is at least comparable to our linear analysis, we would have enough power to find large differences (>8%) in the majority of probes. However, we did not observe such large differences. This power calculation also indicates that we may have missed smaller differences. Therefore, meta-analyses are required to increase statistical power and investigate whether smaller differences in DNA methylation profiles are clinically relevant. Thus far, other studies assessing response to MTX have been conducted using the 450k array. In principle, such studies could be meta-analyzed with our study, however, challenges when combining 450k and EPIC array results exist. Not all probes have been shown to replicate well across the two platforms and several probes are not common to the two arrays [26]. Also, differences in cell types used for the experiments complicate combining studies. Therefore, more studies using the EPIC array in PBMCs are required prior to perform meta-analysis in order to assess whether smaller mean differences are related to response to MTX (combination) therapy. Besides, pyrosequencing could be performed to externally validate the top hits.

Strengths of this study are that it was performed in a prospective cohort where subjects received controlled treatment of similar dosages of MTX (combination) therapy. Despite that the majority received combination therapy, which could potentially influence the outcome (ΔDAS28), no significant differences in ΔDAS28 between treatment groups were observed. Moreover, erythrocyte methotrexate-polyglutamate levels at 3 months were quantified in all patients [15, 27]. In the majority of the samples (66/68), MTX polyglutamate levels were present, supporting treatment compliance. Another strength is that results were acquired from PBMCs consisting of monocytes and lymphocytes but not granulocytes. This is important, as the methylome of granulocytes is very different compared to that of cells of the lymphoid lineage [19]. The downside of using PBMCs is that it is still a cell mixture and that it is more labor intensive to extract them first from whole blood. However, in this study we assessed the cell type composition using the Houseman method [13] and included cell type percentages as covariates. Furthermore, RA is an infiltrating disease; hence for future studies DNA methylation statuses from leukocytes in the synovial fluid may be more predictive in relation to disease activity. Another weakness is that we may have missed small differences due to the limited group size. Furthermore, what could have influenced the relationship between DNA methylation and DAS28 was the presence of anti-citrullinated protein antibody (ACPA) [20]. However, in our study 80% of the patients were positive for ACPA, accordingly we did not correct for ACPA positivity in the models.

In conclusion, we performed the first DNA methylation association analysis using the Illumina MethylationEPIC array to test for treatment response in naïve early rheumatoid arthritis patients. We did not observe genome-wide significant DMPs or DMRs in relation to changes in DAS28 over the first 3 months of treatment. Larger studies are required to demonstrate or rule out the use of DNA methylation sites as predictive marker for response to MTX. Potential biomarkers could be combined with other clinical and laboratory predictors to improve prediction to response and personalize treatment in early RA.

## Supporting information

S1 Fig QQ-plot of DMP analysis with selected probe set. (TIF)

S2 Fig Manhattan plot of DMP analysis with selected probe set. Low variance probes (baseline methylation SD<0.02) and probes on sex chromosomes were excluded. Associations were adjusted for age, gender, smoking and cell type ratios.
(TIF)

S3 Fig Look up study of CpG finding from Glossop et al. Dashed horizontal line represents previously established cut-off value. Response was categorized in non−responders (n = 10), moderate responders (n = 28) and good responders (n = 31) according to the EULAR criteria at 3 months.
(TIF)

S1 Table Top Gene Ontology (GO) terms from pathway analysis of 1000 most significant probes in DMP analysis with full probe set. BP = biological process, CC = cellular component, MF = molecular function.
(TIF)

S2 Table Top DMPs from selected probe set and the association with ΔDAS28. Effect = beta coefficient; the change in DAS28 corresponding to an increase in methylation of 1%. *Genes annotated using the GREAT tool (Human GRCh37 UCSC hg19). Chr = chromosomes, TSS1500 = 200–1500 base pairs from transcriptional start site (TSS). Relation to gene was obtained from the Illumina annotation file version B4.
(TIF)

S3 Table Top 10 DMR results with selected probe set. Chr = chromosome, distance from transcriptional start site (TSS) is reported in ase pairs. N = number of probes, b = change in DAS28 upon 1% difference in baseline methylation.
(TIF)
