# Supplementary figures and images for: Epigenome wide association study of response to methotrexate in early rheumatoid arthritis patients
Source: PLoS One. 2021 Mar 10;16(3):e0247709. doi: 10.1371/journal.pone.0247709 (PMC7946177; doi:10.1371/journal.pone.0247709)

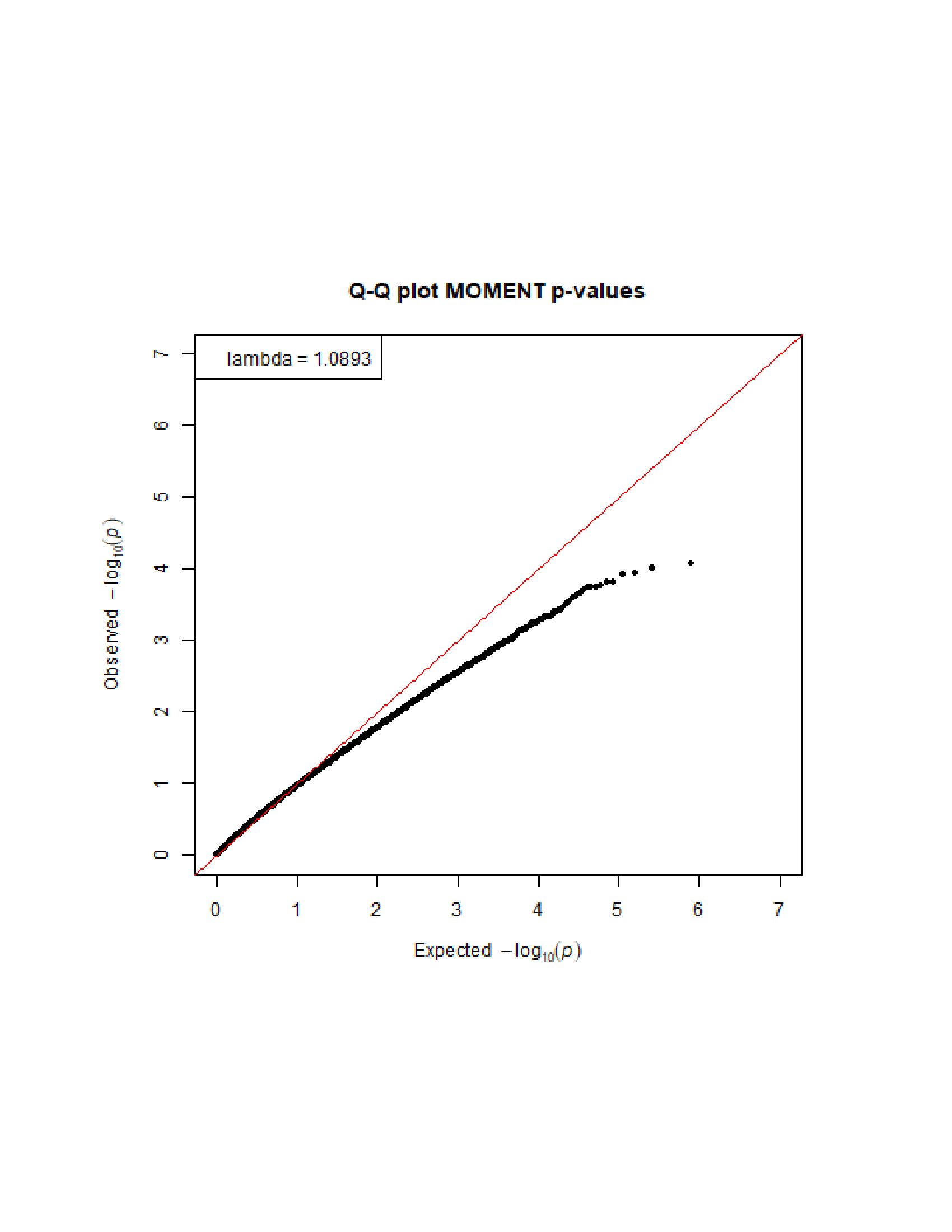

Supplement: S1 Fig — (TIF) [file pone.0247709.s001.tif]

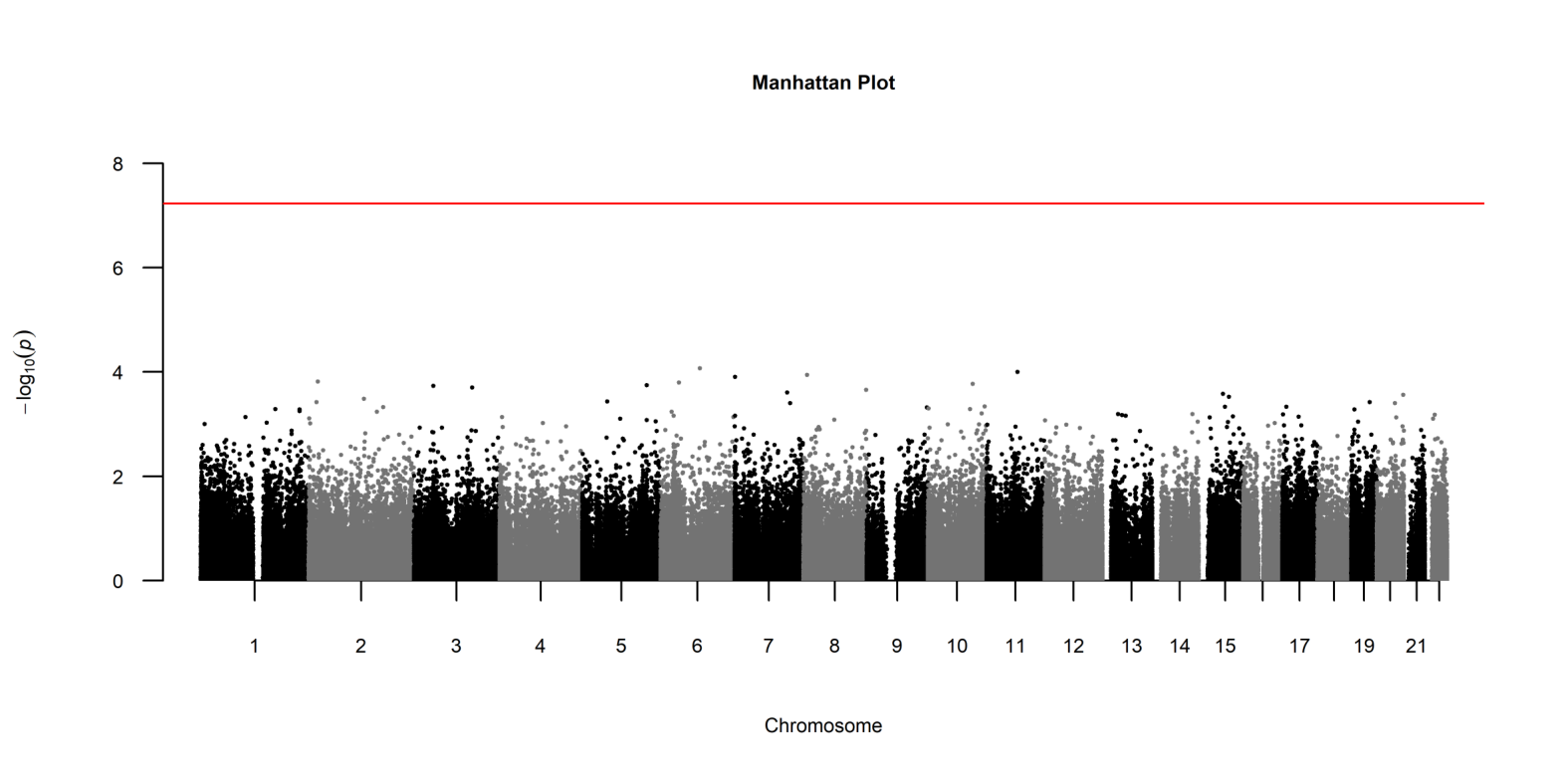

Supplement: S2 Fig — Low variance probes (baseline methylation SD<0.02) and probes on sex chromosomes were excluded. Associations were adjusted for age, gender, smoking and cell type ratios. (TIF) [file pone.0247709.s002.tif]

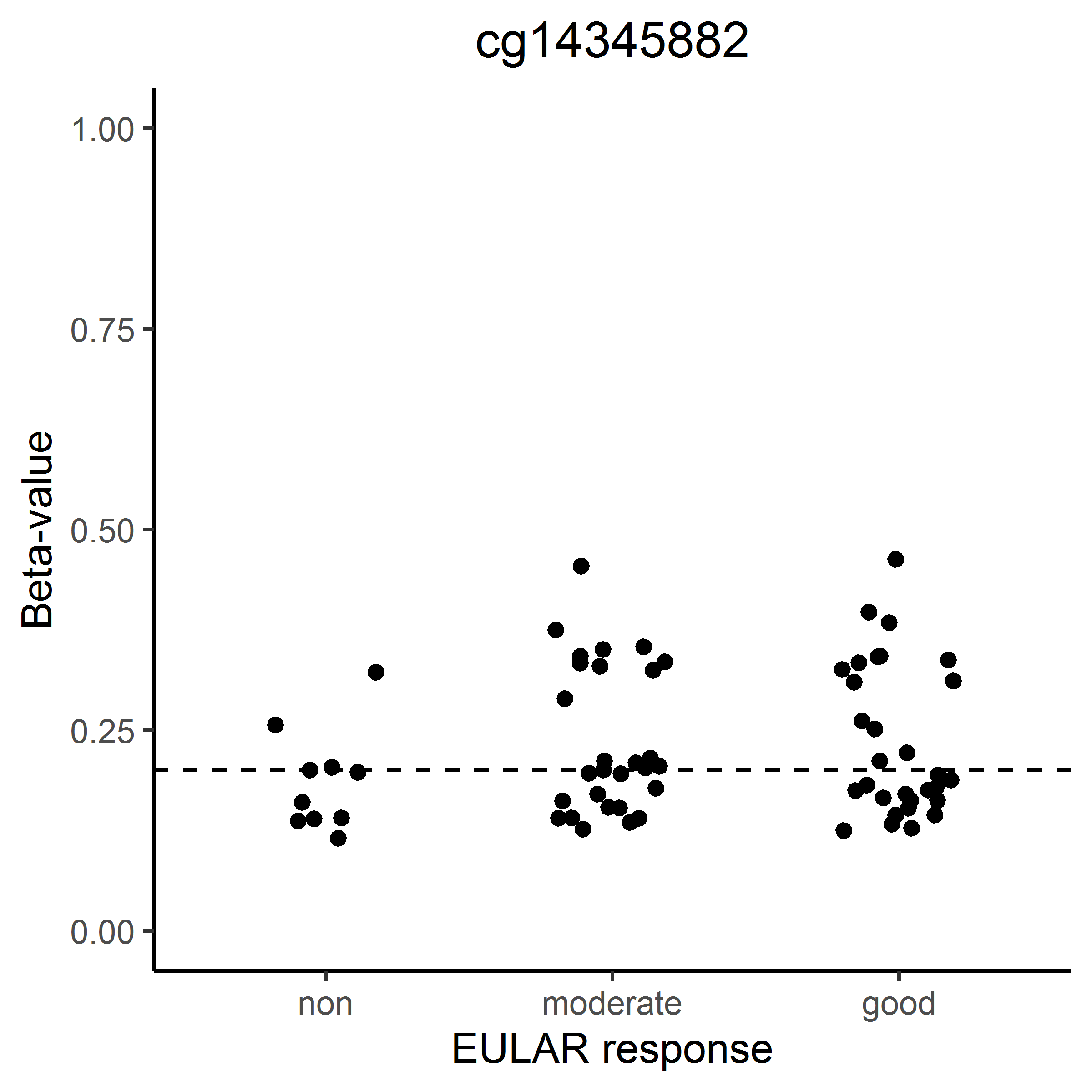

Supplement: S3 Fig — Dashed horizontal line represents previously established cut-off value. Response was categorized in non−responders (n = 10), moderate responders (n = 28) and good responders (n = 31) according to the EULAR criteria at 3 months. (TIF) [file pone.0247709.s003.tif]

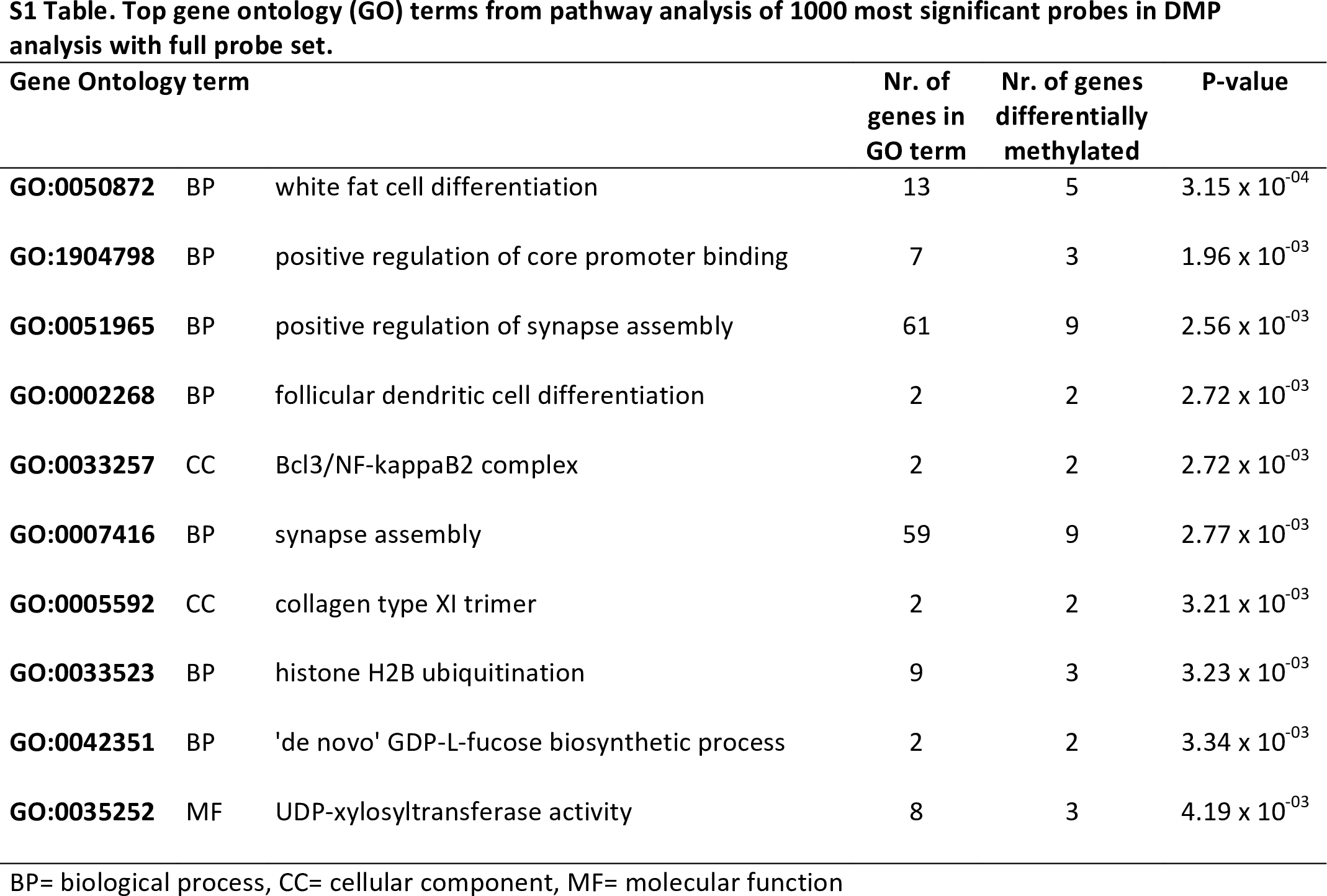

Supplement: S1 Table — BP = biological process, CC = cellular component, MF = molecular function. (TIF) [file pone.0247709.s004.tif]

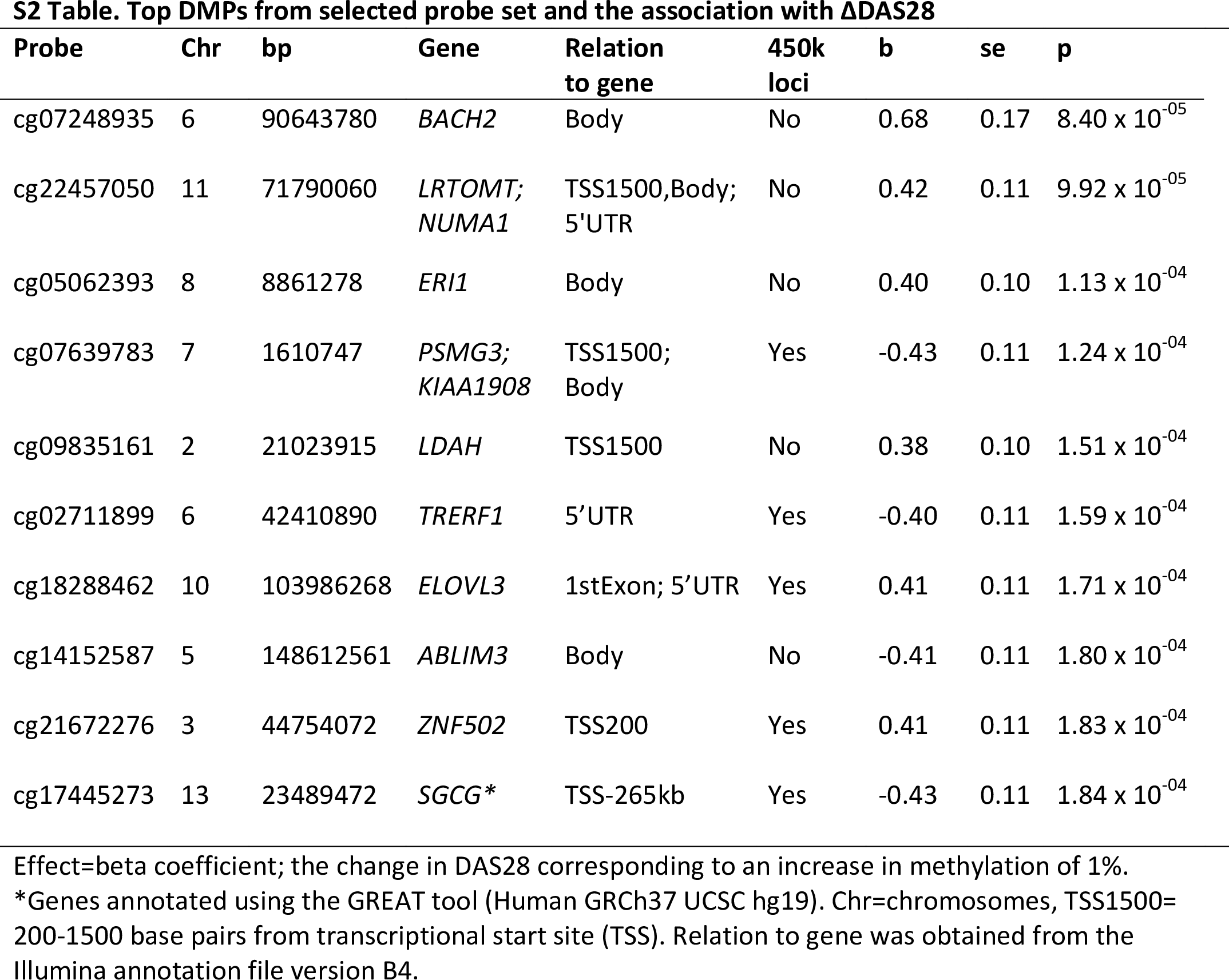

Supplement: S2 Table — Effect = beta coefficient; the change in DAS28 corresponding to an increase in methylation of 1%. *Genes annotated using the GREAT tool (Human GRCh37 UCSC hg19). Chr = chromosomes, TSS1500 = 200–1500 base pairs from transcriptional start site (TSS). Relation to gene was obtained from the Illumina annotation file version B4. (TIF) [file pone.0247709.s005.tif]

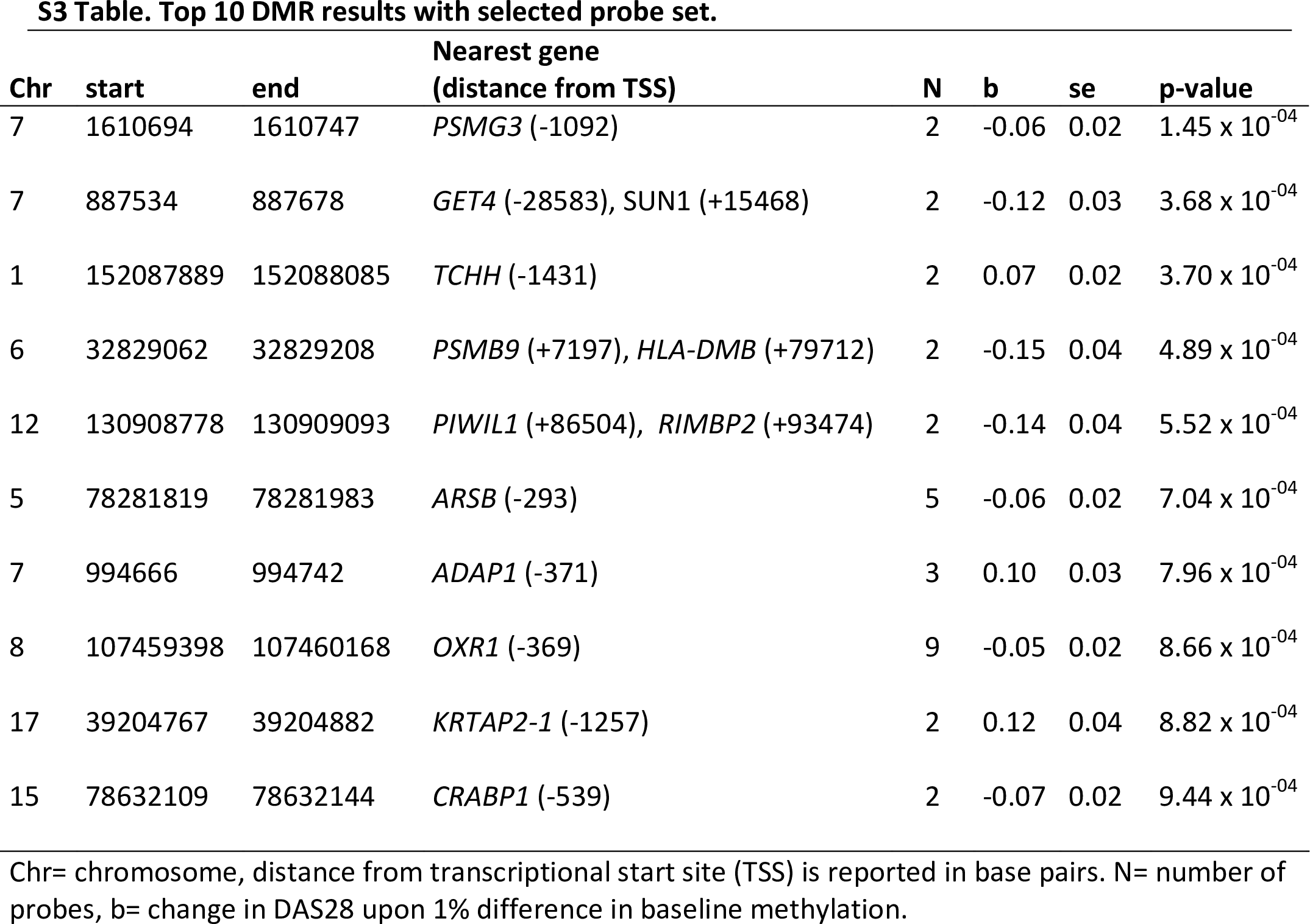

Supplement: S3 Table — Chr = chromosome, distance from transcriptional start site (TSS) is reported in ase pairs. N = number of probes, b = change in DAS28 upon 1% difference in baseline methylation. (TIF) [file pone.0247709.s006.tif]
